# Tissue Damage in Radiation-Induced Oral Mucositis Is Mitigated by IL-17 Receptor Signaling

**DOI:** 10.3389/fimmu.2021.687627

**Published:** 2021-06-17

**Authors:** Jessica Saul-McBeth, John Dillon, Aaron Lee, Dylan Launder, Jacqueline M. Kratch, Eanas Abutaha, Alexandria A. Williamson, Allen G. Schroering, Grace Michalski, Priosmita Biswas, Samuel R. Conti, Amol C. Shetty, Carrie McCracken, Vincent M. Bruno, E. Ishmael Parsai, Heather R. Conti

**Affiliations:** ^1^ Department of Biological Sciences, University of Toledo, Toledo, OH, United States; ^2^ Department of Radiation Oncology, Division of Medical Physics, The University of Toledo, Toledo, OH, United States; ^3^ Department of Surgery, The University of Toledo, Toledo, OH, United States; ^4^ Institute for Genome Sciences, University of Maryland School of Medicine, Baltimore, MD, United States

**Keywords:** oral mucositis (OM), inflammation, interleukin-17, oral mucosa, healing

## Abstract

Oral mucositis (OM) is a treatment-limiting adverse side effect of radiation and chemotherapy. Approximately 80% of patients undergoing radiotherapy (RT) for head and neck cancers (HNC) develop OM, representing a major unmet medical condition. Our understanding of the immunopathogenesis of OM is limited, due in part to the surprising paucity of information regarding healing mechanisms in the oral mucosa. RNAseq of oral tissue in a murine model that closely mimics human OM, showed elevated expression of IL-17 and related immune pathways in response to head and neck irradiation (HNI). Strikingly, mice lacking the IL-17 receptor (IL-17RA) exhibited markedly more severe OM. Restoration of the oral mucosa was compromised in *Il17ra^−/−^* mice and components associated with healing, including matrix metalloproteinase 3, 10 and IL-24 were diminished. IL-17 is typically associated with recruitment of neutrophils to mucosal sites following oral infections. Unexpectedly, in OM the absence of IL-17RA resulted in excessive neutrophil recruitment and immunopathology. Instead, neutrophil activation was IL-1R-driven in *Il17ra^−/−^* mice. Blockade of IL-1R and depletion of neutrophils lessened the severity of damage in these mice. Overall, we show IL-17 is protective in OM through multiple mechanisms including restoration of the damaged epithelia and control of the neutrophil response. We also present a clinically relevant murine model of human OM to improve mechanistic understanding and develop rational translational therapeutics.

## Introduction

Oral mucositis (OM) is one of the most common non-hematological side effects of radiotherapy (RT) and/or chemotherapy for head and neck cancers (HNC), characterized by tissue injury of the oral mucosae (1). OM is painful and associated with nutritional deficiency, thus resulting in a large economic burden due to costs and clinical risks associated with pain management and liquid diet supplementation (2). In the 100 days following the initial development of OM, the risk of mortality increases 3.9-fold (3). During OM the loss of oral mucosal integrity, imbalance of the oral flora and hyposalivation leads to increased susceptibility to oral infections, including oropharyngeal candidiasis (OPC) and herpes simplex virus (HSV), pronounced sensitivity to dental caries, gingivitis and periodontitis (3–5). The current therapeutic options for OM are largely ineffective and management relies on lessening symptoms, not prevention (6–9). Ultimately, severe OM can lead to increased hospitalizations and the use of feeding tubes, which may interrupt or alter cancer therapy leading to worse tumor outcomes (2, 6, 10–12).

The stages of OM in both humans and mice include initiation, primary damage with signal amplification, ulceration, followed by healing and fibrosis (2, 13). Ionizing radiation initiates OM through toxicity to basal epithelial, submucosal, and endothelial cells (3, 14). Cells with extensive DNA damage produce reactive oxygen species (ROS) that accumulate in tissue (11). Injured cells also release damage-associated molecular patterns (DAMPs) that bind receptors to initiate inflammatory signaling cascades (12). Eventually, repair and healing occurs *via* signaling from the extracellular matrix (ECM) and anti-inflammatory cytokines (2). It is understood that the progression of OM requires activation of NF-kappaB which induces transcriptional expression of pro-inflammatory mediators, such TNF-α, IL-1, and IL-6 (2, 11, 15). Sustained expression of these cytokines perpetuates tissue damage, resulting in the loss of membrane integrity and development of oral ulcers (13). While the clinical progression of OM is described, the intricate signaling networks involved in the progression and resolution have not been fully elucidated (8, 9, 11). Indeed, there is a general paucity of information regarding the immunology of the oral mucosa, highlighting a need to better understand these processes.

Interleukin-17 (IL-17) is a proinflammatory cytokine that plays diverse roles in oral health and disease. For example, IL-17 is protective against acute bone loss caused by gingivitis-related bacteria (16). However, in chronic periodontitis, IL-17 promotes inflammation-induced bone loss (17). Furthermore, due to the proliferative and inflammatory properties of IL-17, it is implicated in tumorigenesis in several cancers, including esophageal cancer (18, 19). IL-17 also supports tongue squamous cell carcinoma formation (20, 21). In other tissue compartments, the roles for IL-17 in damage and healing are not straightforward either. IL-17 protects the intestinal epithelium through maintenance of tight junctional proteins and stimulating epithelial cell proliferation (22, 23). Whereas, inhibition of IL-17 or IL-17RA leads to a weaker intestinal epithelial barrier and higher incidence of severe disease in the gut (22, 24). When considering the skin though, the proliferative capacity of IL-17 can be damaging during psoriasis, yet advantageous to tissue repair during injury, likely *via* similar effects on the epithelial layer (25–27).

Since IL-17 has distinct roles in tissue repair and maintenance it was important to determine how the cytokine functions during the severe ulceration associated with head and neck irradiation (HNI). We focused on whether IL-17RA was protective or pathogenic during oral damage and healing associated with radiation. We approached this by exposing *Il17ra^−/−^* mice to HNI-induced OM. Here, we provide evidence that IL-17RA signaling is beneficial during OM by promoting tissue regeneration and unexpectedly dampening the neutrophil response. In the absence of IL-17RA, other mediators, including IL-1, were dysregulated leading to excessive inflammation and tissue damage. Overall, while IL-17/IL-17RA are potential targets in HNC, care must be taken in establishing therapeutic strategies in patients who develop OM during radiation treatment.

## Materials and Methods

### Mice

Mice were acquired by materials transfer agreement (MTA) with Amgen (*Il17ra* ^−/−^) (28). In all experiments, age- and gender-matched littermate controls or WT controls (Jax Inc.) were used. All mice were housed with food and water ad libitum under a 12-hour dark/light cycle in a specific pathogen-free facility at the University of Toledo. All animals were used in accordance with the protocol reviewed and approved by the Institutional Animal Care and Use Committee and in accordance with guidelines from the National Institutes of Health, the Animal Welfare Act, and U.S. Federal Law.

### Radiation-Induced OM

To expose mice to HNI and induce OM, mice were immobilized using an anesthesia protocol approved by the Department of Laboratory Animal Research at the University of Toledo. Mice were aligned in a custom-made Polystyrene phantom with the aid of the University of Toledo Department of Radiation Oncology staff, using the Linear Accelerator’s (Linac’s) field light to assure only the head received radiation. A one cm tissue-mimicking super flab bolus material was used to assure the distribution of the dose at a 99–100% level was in the entire depth intended for treatment. This assured over 99% dose to the surface of the skin and at the distal depth which was on average 1.5 cm. The energy used for this experiment was a 6 MeV electron beam and monitor units were calculated to deliver 22.5 Gy at the rate of 1,000 cGy/min in a single fraction. Based on characteristics of the 6 MeV electron profile and realizing that 50% isodose line was at the edge of the field, the mouse’s head and tongue area received 80 to 85% of the nominal 22.5 Gy amounting to 18–19 Gy. The effective delivered dose throughout this manuscript should then be taken as 80 to 85% of what is presented as the nominal dose. The custom manufactured jigs at the head of the Linac were used to align the body such that only the head was exposed, and the body protected from the radiation beam. Following irradiation, animals were removed from the jig, housed in a climate and light/dark controlled environment, and allowed free access to food and water. Animals were monitored daily for changes in weight and activity.

### Macroscopic and Histopathologic Examination

Tongues were rinsed with PBS and stained with 1% toluidine blue for 2 min, followed by washing with acetic acid for 30 s to reveal ulcerative lesions. The percentage of toluidine blue-positive areas was calculated using ImageJ software and % damage quantified by the area of toluidine blue positive area/surface area of whole tongue ∗ 100. Tissues were formalin-fixed, paraffin-embedded, and sectioned at a thickness of 5 µm. Ulcer size, mucosal thickness, and cellular infiltrate were measured in H&E-stained tissue using a Bio-Tek Cytation 5 automated microscope (Bio-Tek) and equipped with image-capturing software by The University of Toledo Advanced Microscopy & Imaging Center (Toledo, OH). Investigators analyzing staining of tongues were blinded to treatment and mouse cohort.

### Immunohistochemistry

Tissues were formalin-fixed, paraffin-embedded, and sectioned at 5 µm. Slides were dehydrated with xylene and ethanol gradient, and antigen retrieval and blocking performed. Sections were further labeled with MPO (R&D Systems, Minneapolis, MN) or ki67 (Cell Signaling Technology, Danvers, MA). Secondary biotinylated antibody was applied, and slides incubated at room temperature for 1 h. Signals were detected using Sigma Fast tablets to make the DAB solution (Sigma Aldrich, St. Louis, MO) and the reaction stopped by placing slides in TBS. For IL-1α, MMP9, and TIMP2 IHC was performed on paraffin sections using avidin-biotin-peroxidase complex (streptavidin–biotin labeled method) with the Cell and Tissue staining kit (R&D Systems, Minneapolis, MN). The manufacturer’s protocol was followed. The antibodies for IL-1α, MMP9, and TIMP2 were purchased from R&D Systems, Inc. (Minneapolis, MN).

### ELISA

Fresh or frozen (−80°C) tongue tissue was submerged in cold sterile saline and homogenized by GentleMACS Dissociator (Miltenyi Biotec) in lysis buffer containing: 50 mM potassium phosphate buffer pH 6.0 and 0.5% HTAB. Samples were lysed further by 3× freeze thaw at 37°and centrifuged at 10,000×*g* 4° for 15 min. Supernatants were collected and protein concentration determined by BCA (Thermo Fisher Scientific). Following protein determination, IL-1β was semi-quantitatively measured by enzyme-linked immunosorbent assay (R&D Systems) according to manufacturer’s protocol. Assay was performed in biological triplicate in technical duplicate.

### Complete Blood Count

EDTA anti-coagulated blood samples from cardiac puncture were used to obtain a complete blood count with an insight V5 Hematology Analyser (Woodley Equipment, Bolton, Lancashire).

### Real-Time Reverse Transcription-PCR

Total RNA was extracted using TRI reagent (Sigma-Aldrich, St. Louis, MO) and RNA (1 µg) reverse-transcribed by High-Capacity cDNA RT kit (Thermo Fisher Scientific, Waltham, MA) at 25° for 10 min, 37°C for 120 min, followed by 85° for 5 min. Quantitative PCR was performed using PowerUp SYBR green Master Mix and a Quant Studio 3 detection system (Applied Biosystems, Waltham, MA), as specified by the manufacturer. The crossing point was defined as the maximum of the second derivative from the fluorescence curve. For quantification, we report relative mRNA expression of specific genes using the 2^−ΔCT^ method and used GAPDH housekeeping gene for normalization. Primers that were made in house are shown in **Supplementary Table 1**, otherwise primers were obtained from QuantiTect (QIAGEN, Germantown, MD). Assays were performed in biological triplicate in technical triplicate.

### RNA Sequence Analysis

RNA-seq libraries (strand-specific, single end) were generated from total tongue RNA by using a NEBNext Ultra II Directional RNA Library Prep kit (New England BioLabs, Ipswich, MA). Fifty nucleotides of the sequence were determined from one end of each cDNA fragment using the HiSeq platform (Illumina). Sequencing reads were aligned to the UCSC (University of California, Santa Cruz) mouse reference genome (mm10,GRCm38.75) using HISAT (29), and alignment files were used to generate read counts for each gene. Statistical analysis of differential gene expression was performed using the DEseq package from Bioconductor (30). A gene was considered differentially expressed if the FDR value for differential expression was <0.01. The RNA-seq analysis was performed in biological triplicate.

Enrichment and pathway analyses of the differentially expressed genes were performed using the Upstream Regulator Analytic and the Diseases and Function analytic from the Ingenuity Pathway Analysis software (Ingenuity Systems; http://www.ingenuity.com). This software assesses the overlap between experimentally derived gene lists and an extensively curated database of target genes for each of several hundred known regulatory proteins and pathways. It then uses the statistical significance of the overlap and the direction of the differential gene expression to make predictions about activation or repression of pathways.

### Antibody Treatment in OM Model

Doses of anti-IL-1R (anti-Ly6G (BioXcell), and anti-G-CSF (R&D Systems) neutralizing antibodies were based on previous studies (31–33). Isotype control antibodies dose and administration schedules are included (**Supplementary Table 2**). Mice were treated i.p. on days 0, 2, 4, 6, and 8 of the experiment with antibodies directed against IL-1R (300 µg/mouse or IgG isotype control (BioXcell) (300 µg/mouse), in age- and sex-matched controls. For the anti-Ly6G (150 µg/mouse) and anti-G-CSF (10 µg/mouse) mice were treated i.p. with both antibodies and controls (R&D Systems) on d7, followed by daily treatments of G-CSF on d8, 9, and 10 of the experiment. Mice were treated with α-IL-17A or isotype control (BioXcell) at a 150 µg/mouse on days 8 and 10 post irradiation. On day 11 following HNI, tongues were harvested and assessed for tongue damage by toluidine blue+ staining.

### Flow Cytometry

Tongue tissue was mechanically homogenized in RPMI 1640 media then incubated at 37°C for 42 min on a GentleMACS Dissociator (Miltenyi Biotec) using tissue dissociation kits (Miltentyi Biotec). Resulting solution was passed through a cell strainer for single-cell suspensions. After brief centrifugation, cells were reconstituted with PBS supplemented with 2% FBS and 2 mM EDTA. Some 2 × 10^6^/ml viable cells were obtained by staining with Trypan blue and counting on a hemocytometer. For analysis of immune cells, an initial incubation of CD16/CD32 Fc Block (BD Biosciences) was followed by staining with the following antibodies, all from BioLegend (San Diego, CA): CD11b-PerCP/Cyanine5.5 (M1/70), GR-1-APC (RB6-8C5), F4/80-APC/Cyanine7 (BM8), and I-A/I-E-BV510 (M5/114.15.2).

For analysis of apoptosis, separate cell suspensions were reconstituted in Annexin V Binding Buffer followed by incubation with Annexin V-PE/Cy7 antibody and Propidium Iodide (Biolegend, San Diego, CA), for 10 min before flow cytometric analysis. Flow cytometry was performed on an LSRFortessa (BD Bioscience, San Jose, CA) and analyzed with FlowJo (BD Bioscience, San Jose, CA). For flow analysis lymphocyte and myeloid populations were gated out, and the resulting epithelial cell populations were further analyzed (**Supplementary Figure 3**).

### Data Analysis

Data was analyzed on Prism (Graphpad V8.4.3). ELISA data is presented as one-way ANOVA with Tukey’s *post hoc* analysis. Flow cytometry was analyzed by day with Student’s *t* test and is presented as the mean ± SEM. Tongue damage data is presented as geometric mean ± SEM and analyzed by ANOVA with Tukey’s *post hoc* analysis or Student’s *t* test for analysis between two groups. PMN quantification was analyzed by ANOVA with Tukey’s *post hoc* analysis. Gene expression data is presented as mean ± SEM analyzed by Kruskal–Wallace test with Dunn’s multiple comparisons or ANOVA with Tukey’s *post hoc* analysis. Normality was evaluated *via* Shapiro–Wilk tests. Each symbol represents one mouse unless indicated. *P <0.05, **P <0.01, ***P <0.001, and ****P <0.0001.

## Results

### HNI Induces an IL-17-Related Transcriptional Profile During OM

**Figure 1:**
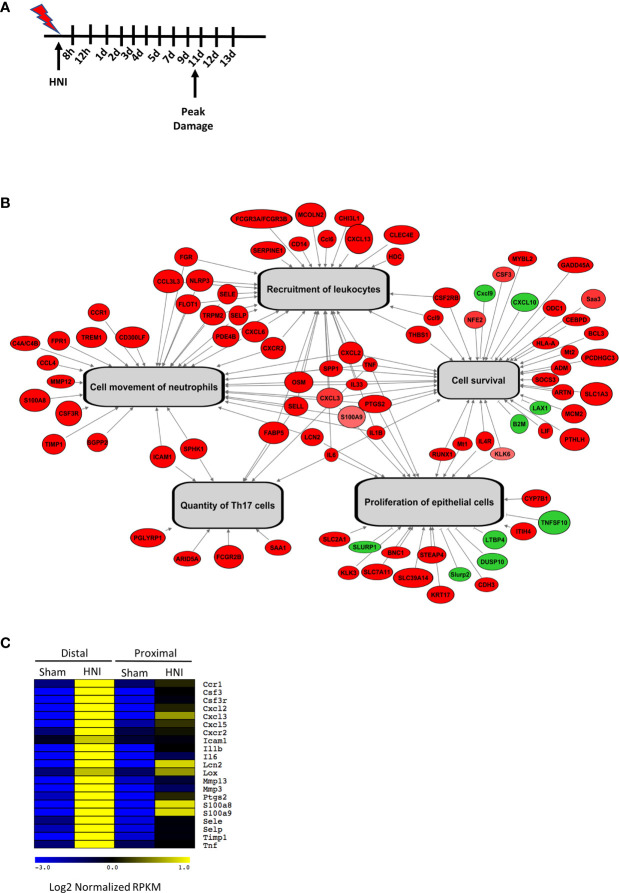
Targeted HNI induces OM in mice. **(A)** Model of HNI-induced OM. Mice received no radiation (sham) or HNI on D0, and tongues were harvested at various time points. **(B)** Schematic analysis of select biological functions predicted to be activated in response to HNI. Genes colored in red have HNI-induced expression and genes labeled in green have HNI-repressed expression. Gray arrows depict biological functions that are predicted by our analysis to be activated. **(C)** Comparison of IL-17RA regulated genes between sham and irradiated mice in the distal portion of the tongue.

Sensitivity to OM is dependent on many factors including treatment type, dose rate, total dose, and volume irradiated. Most patients with HNC are treated with external beam radiotherapy using a relatively newer modality known as Intensity Modulation Radiotherapy (IMRT). This can only be implemented using complex treatment planning algorithms that can account for behavior of primary and scatter radiation in areas of heterogeneity (34, 35). In addition, these treatment planning systems contain optimization routines for inverse planning to achieve optimal dose to the target volume while sparing normal tissue. All of these newer features have been instrumental in reducing the severity of OM in recent years, but we still consider this as a major side effect of radiation for HNC patients that needs to be dealt with (35). In order to model OM, we used a single-dose of radiation targeting the head and neck regions of mice using a clinical linear accelerator capable of IMRT delivery. To define the stages of OM through healing, mice were irradiated and tongue tissue harvested daily for 13 days (**Figure 1A**). Irradiated mice presented with symptoms typically associated with human OM, including damage and overt lesions on the tongue. A dose of 22.5 Gy caused damage to the oral mucosa that was evident on Day 8 (~4% of tissue toluidine blue+), peaked on Day 11 (~23% of tissue, with complete healing by Day 15 (0% of tissue), which is similar to the stages of OM in humans (8, 36, 37) (**Supplementary Figure 1A**). In order to understand the immune components responsible for signal amplification and ulceration in the tongue tissue during OM, we performed RNA sequencing (RNA-Seq) analysis of mRNA from tongue tissue of non-irradiated sham mice and mice exposed to 22.5 Gy, harvested on Day 11 during peak damage (n = 3). The sequencing profiles showed that 988 genes exhibited a change in expression (FDR <0.01) in at least one of the HNI versus sham comparisons (**Supplementary Tables 3**, **4**). Next, we used Ingenuity Pathway Analysis (IPA) to identify pathways activated during OM. Processes involved in epithelial cell survival and proliferation were enriched during peak damage. The most differentially expressed genes were also involved in immune functions such as global recruitment of immune cells (p=2x10^-18^), movement of polymorphonuclear cells (PMNs) (p = 4 × 10^−11^), and development of Th17 cells (p = 4 × 10^−6^) (**Figure 1B**).

**Figure 2:**
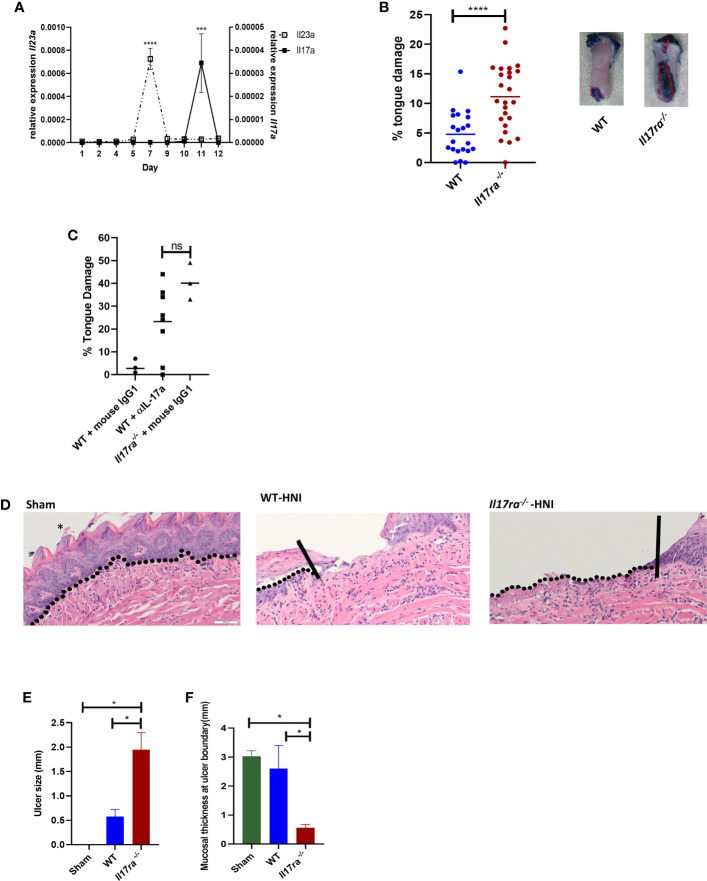
IL-17 is induced and is protective following HNI **(A)** Expression kinetics of *Il23 *and *Il17a *following HNI assessed by qPCR. Results pooled from at least three experiments analyzed by ANOVA with Tukey’s *post hoc*. **(B)** Quantification of toluidine blue staining by determining the surface area of the tongue positive for blue staining compared to the total surface area of tongue on each mouse on day 11 post radiation. The staining on the ventral side of the tongue was not included in the quantification since this included the excision site. Red dotted lines represent the surface area of a lesion as determined for all tongues. Data pooled from at least three experiments. Analyzed by Student’s *t* test. **(C)** Mice were treated on days 8 and 10 with α-IL-17A or mouse IgG1 (150 µg) and damage quantified on day 11 (n = at least three per group). Control animals received appropriate isotype controls (**Supplementary Table 2**). Tongue damage analyzed by ANOVA with Tukey’s. **(D)** Hematoxylin–eosin staining of mouse tongues D11 following radiation. Asterisk denotes papillae. The vertical lines in the images of tongues highlight the ulcer boundary and the dotted lines represent the epithelial-stromal boundary. **(E, F)** Quantification of lingual ulcer size and mucosal thickness (N= at least 5 per group). Data represents as least three pooled experiments. Analyzed by ANOVA with Tukey’s *post hoc*. Scale bars: 50 µM. Data shown as means ± SEM, (*P < 0.05, ***P < 0.001, ****P < 0.0001. NS is not significant.

Further analysis of genes differentially expressed in mice with OM showed many immune components related to the IL-17 signaling pathway were activated after HNI, with stronger induction in the distal portion of the tongue where damage predominated (**Figure 1C** and **Supplementary Figure 1B**). These targets included chemokines, cytokines, signaling molecules, AMPs, and proteases that are regulated by IL-17RA in the context of other diseases (38). Since IL-17-related genes were induced during peak damage, we next determined the expression kinetics of both *Il17a* and *Il23a* throughout the stages of OM. After HNI, *Il23a* expression peaked on Day 7 followed by a rapid loss of expression by Day 9. Induction of *Il23a* was followed by *Il17a* expression, which was detected after Day 9 and increased by Day 11, the time point at which WT mice presented with severe ulcerative damage. The levels of *Il17a* dropped by Day 12 as healing commenced (**Figure 2A**). Taken together these data indicate that the IL-23/IL-17 pathway is activated following HNI, and IL-23 expression preceding IL-17 is predicted, as it is understood to be upstream of IL-17 production.

### IL-17RA Signaling Protects Against Ulcerative Damage and Epithelial Loss Following HNI

In order to determine if IL-17RA is protective or pathogenic during the most severe phase of OM, WT and *Il17ra^−/−^* mice were subjected to HNI and damage assessed. Larger ulcerative lesions were detected in *Il17ra^−/−^* mice (~11% of tongue) on Day 11 compared to WT mice (~5% of tongue) (**Figures 2B, D** **)**. In addition to the increased surface area of damage in the *Il17ra^−/−^* mice (**Figure 2E**), lesions were deeper (1.8 mm *vs*. 0.6 mm) and the adjacent tissues were thinner (0.5 mm *vs*. 1.7 mm) than those in WT mice during peak damage (Day 11) (**Figure 2F**). Next, we treated mice with α-IL-17A or mouse IgG1 isotype control to determine if therapeutic blockade of IL-17 rendered mice more susceptible to OM. There were no differences in damage between mice treated with α-IL17A or *Il17ra^−/−^* mice (**Figure 2C**). Based on these findings, IL-17A/IL-17RA appear protective in HNI-induced damage to the oral mucosa.

### IL-17RA Promotes Cell Survival and Proliferation During OM

**Figure 3:**
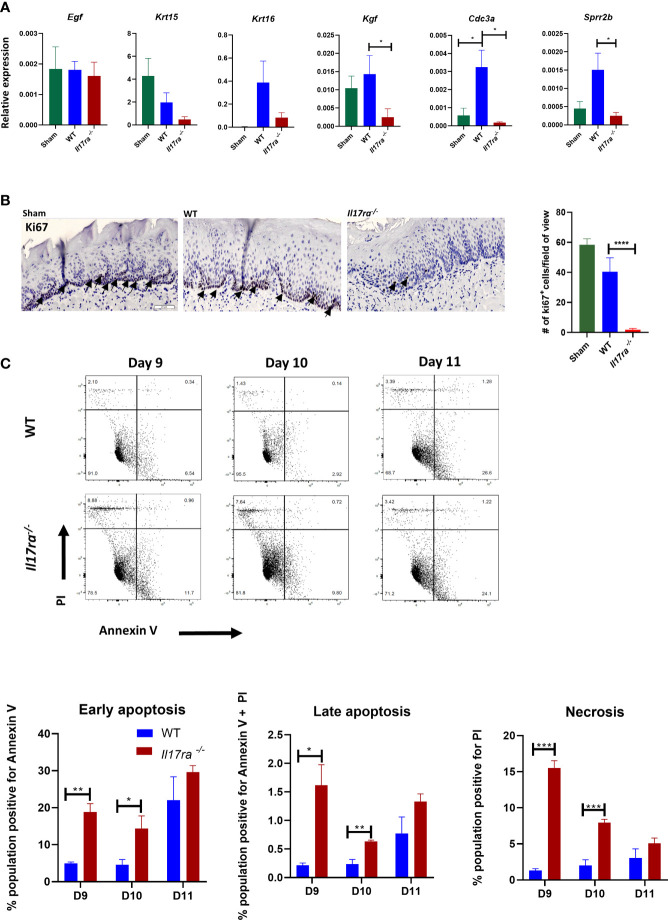
In the absence of IL-17RA there is enhanced apoptosis and reduced proliferation **(A)** Expression differences relative to GAPDH of genes related to healing. N = at least five mice/group. *Kgf* and *Cdc3a* analyzed by Kruskal–Wallace with Dunn’s multiple comparisons test, *Sprr2b* analyzed by ANOVA with Tukey’s. Data pooled from at least three experiments **(B)** Proliferation marker Ki-67 staining and quantification in the dorsal portion of the tongue obtained on day 11 following radiation. Scale bar, 100 µM. Arrows represent positive Ki67 staining. Analysis of staining by ANOVA with Tukey’s. **(C)** Apoptotic and necrotic cells analyzed by flow cytometric analysis after Annexin V and PI staining. Data representative of two independent experiments and analyzed by Student’s *t* test between days. Data shown as means ± SEM, (*P < 0.05, **P < 0.01, ***P < 0.001, ****P < 0.0001).

To elucidate the mechanism of IL-17RA-mediated protection, we next determined the role of IL-17 signaling in epithelial repair of the oral mucosa after radiation. Epithelial proliferation contributes to the rate of healing during OM, which is partly dependent on epidermal growth factor (EGF) and keratinocyte growth factor (KGF) (8). While expression of *Egf* was comparable between *Il17ra^−/−^* and WT mice regardless of radiation exposure, markers for early differentiated epithelial cells, keratin-15 (*Krt15*) and keratin-16 (*Krt16*), were reduced in *Il17ra^−/−^* mice, as was *Kgf* (**Figure 3A**), signifying a lack of re-epithelialization (39). Similarly, expression of *Cdc3a*, which is essential for cytokinesis, was reduced in *Il17ra^−/−^* mice. Furthermore, Ki67 staining in tongue tissue revealed fewer dividing cells in the basal stem cell layer of irradiated *Il17ra^−/−^* mice (two cells/field of view) compared to WT mice (40 cells/field of view) (**Figure 3B**). Also, the number of Ki67+ cells in irradiated WT mice was not significantly different compared to WT sham controls (59 cells/field of view). Consistently, transcript levels of components normally expressed during the healing process (*Mmp3, Mmp10, Sprr2*b and *Il24*) were highly induced in WT mice during OM, but were all reduced in *Il17ra^−/−^* mice (**Figures 3A**, **5A**, **Supplementary Figure 2**). IL-24 production in the suprabasal region of the tongue tissue was undetectable in *Il17ra^−/−^* mice compared to WT mice (**Supplementary Figure 2A**). In addition, a major cytotoxic effect of RT is activation of apoptosis and necrosis in the proliferating basal cell layer. Relatedly, *Il17ra^−/−^* mice had more necrotic and early/late apoptotic oral mucosal cells on Days 9 through 11 compared to WT mice (**Figure 3C**). In all, suppression of these contributors to the healing process in the absence of IL-17RA further substantiates the importance of the receptor in protection and restoration of the oral mucosa during HNI-induced OM.

### Enhanced Neutrophil Response in IL-17RA-Deficient Mice

In most disease contexts IL-17 does not act on granulocytes directly, but rather promotes neutrophil recruitment through the induction of chemokines including CXCL1, CXCL2 and CXCL5 and growth factors such as G-CSF in target non-hematopoietic cells. In the oral cavity specifically, mice lacking IL-17RA in the suprabasal oral mucosae (*Il17ra^fl/fl^*-K13Cre^+^) have diminished neutrophil levels, contributing to their high susceptibility to oral candidiasis (40). We set out to determine if mice lacking IL-17RA had the same neutrophil defects during OM caused by RT.

**Figure 4:**
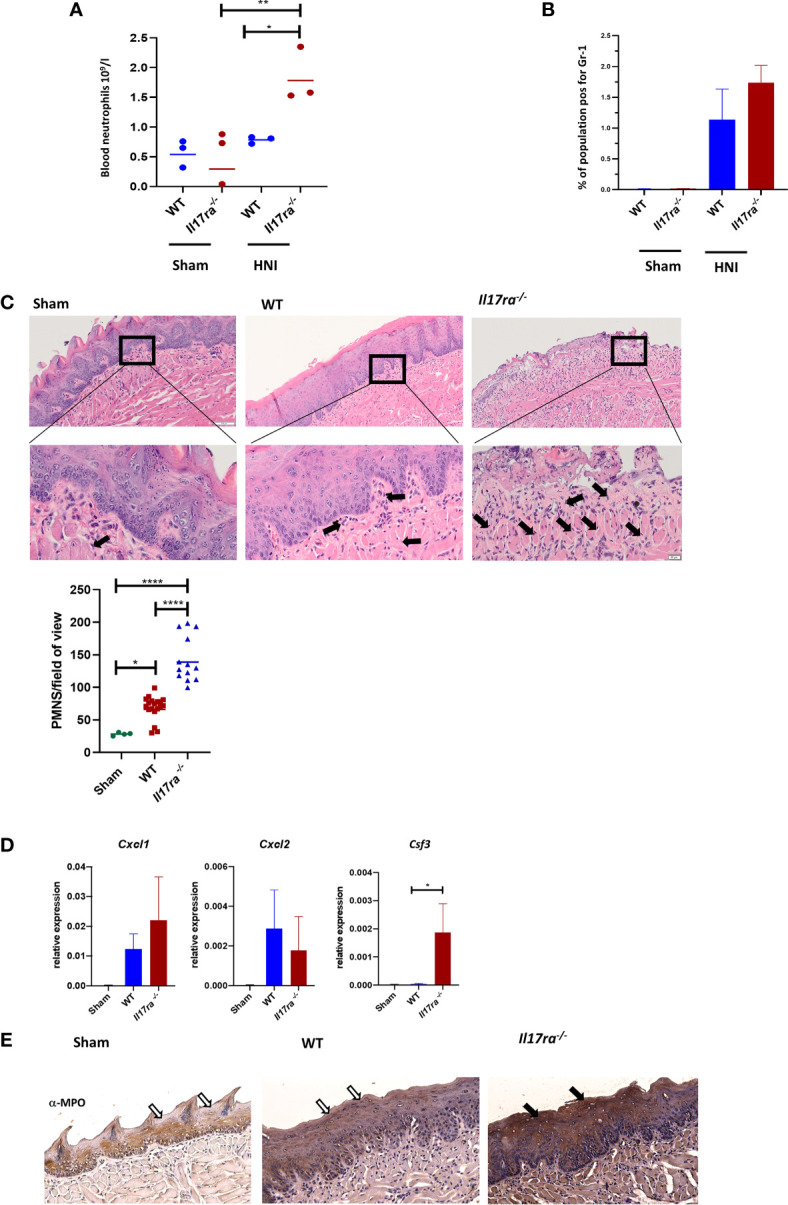
Neutrophil recruitment to the tongue tissue is heightened in the absence of IL-17RA. **(A)** Cardiac puncture was performed to harvest blood from non-irradiated and irradiated mice and number of circulating neutrophils measured on D11. Data is representative of two independent experiments and analyzed by ANOVA with Tukey’s. **(B)** Tongues harvested on day 11 and neutrophil (Gr1+) populations analyzed by flow cytometric analysis and ANOVA with Tukey’s. Data representative of two independent experiments **(C)** Staining by hematoxylin and eosin of sectioned tongues and quantification of neutrophils on day 11 following HNI. Arrows represent positive neutrophil staining in distal tongue at ulcer boundary (N = at least three tongues per group). Scale bar 100 µM (top row) and 20 µM (bottom row). Neutrophil quantification analyzed by ANOVA with Tukey’s *post hoc*. **(D)** Expression of neutrophil chemokines relative to GAPDH on D11 (N = 3–5 per group). *Cxcl1* analyzed by ANOVA with Tukey’s *post hoc*, *Cxcl2* and *Csf3* analyzed by Kruskal–Wallace and Dunn’s multiple comparisons. Data pooled from at least three experiments. **(E)** Representative MPO-staining of ulcer boundary counterstained with hematoxylin. The filled arrow indicates positive MPO staining, while the unfilled arrows show the equivalent areas with absent MPO. Scale bar,100 µM. Data shown as means ± SEM, (*P <0.05, **P <0.01,****P <0.0001).

In patients receiving RT or chemotherapy, the early stages of OM are characterized by neutropenia, and a lack of circulating neutrophils can be a readout for OM severity (41–43). Neutrophils detected in the oral lesion later during the ulceration phase are generally thought to cause damage. The number of neutrophils also increases as microbes gain access to the submucosa, due to loss of barrier integrity (43, 44). By day 11 post-HNI, WT mice had levels of circulating neutrophils comparable to sham mice (**Figure 4A**), suggesting that irradiated mice were not neutropenic at this point. In contrast, the levels of blood neutrophils were elevated in *Il17ra^−/−^* mice. Next, we assessed if neutrophils were recruited to the tongue. Compared to sham, WT and *Il17ra^−/−^* mice had comparable elevated levels of neutrophils in the whole tongue, indicating irradiation induces a neutrophil influx into the tissue at later stages of OM, and this recruitment was surprisingly independent of IL-17RA signaling (**Figure 4B**). We then determined if neutrophils/polymorphonucler cells (PMNs) were localizing to ulcers in the absence of IL-17RA (**Figure 4C**). Unexpectedly again, *Il17ra^−/−^* mice showed significantly more PMNs migrated into the damaged lesions compared to WT mice. While expression of *Cxcl1* and *Cxcl2* were not affected (**Figure 4D**), there was a notable increase in *Csf3* (encodes for G-CSF) in *Il17ra^−/−^* mice compared to WT mice on Day 11 of OM.

Since neutrophil recruitment is normally compromised in mice deficient in IL-17RA we next asked if the neutrophils in the lesion were functionally competent in *Il17ra^−/−^* mice. We measured myeloperoxidase (MPO) levels in tongue, a parameter also commonly used to assess the severity of OM (8, 45, 46). The oral mucosa of irradiated *Il17ra^−/−^* mice had greater MPO production compared to irradiated WT mice on Day 11 aligning with the increased presence of neutrophils in *Il17ra^−/−^* mice (**Figure 4E**).

**Figure 5:**
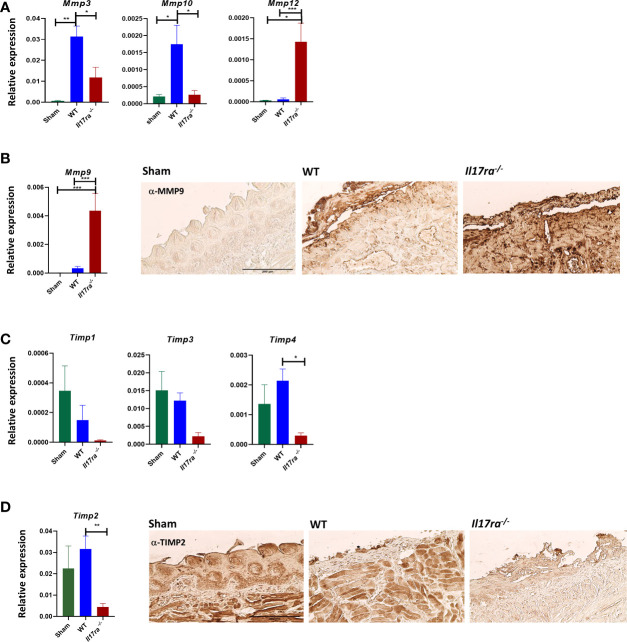
The absence of IL-17RA is associated with dysregulated proteases and inhibitors. **(A, C)** Gene expression relative to GAPDH of proteases and inhibitors on D11 (N = 3–5 per group). N = at least five per group. *Mmp3* and *Mmp10*, and *Timp1,2,3,4* analyzed by ANOVA with Tukey’s *post hoc*, *Mmp12* analyzed by Kruskal–Wallace and Dunn’s multiple comparison test. Data pooled from at least 3 experiments. **(B, D)** IHC for MMP9 and TIMP2 from the ulcer boundary zone in the distal tongue. Scale bar, 200 μM. Data shown as means ± SEM, (*P < 0.05, **P < 0.01, ***P < 0.001).

Neutrophils also activate MMPs through production of elastase (47, 48), so we reasoned that elevated neutrophil-mediated inflammation observed in the *Il17ra^−/−^* mice could be due to dysregulated MMPs and could impact the process of wound healing (49, 50). The increased levels of *Mmp10* and *Mmp3* in the WT tissue during OM were diminished in the *Il17ra^−/−^* tissue (**Figure 5A**). In contrast, *Mmp9*, and *Mmp12* transcripts were elevated, as well as MMP9 protein in the *Il17ra^−/−^* mice compared to sham and irradiated WT mice (**Figures 5A, B**). The skewed expression of MMPs was associated with a decrease in expression of MMP inhibitors, *Timp1*, *Timp2*, *Timp3*, and *Timp4*, and lower TIMP2 production in the *Il17ra^−/−^* mice (**Figures 5C, D**). Collectively, these data indicate that during OM, IL-17RA is not necessary for neutrophil recruitment as it is in other disease settings, nor is IL-17 signaling involved in promoting neutrophil function. Moreover, in the absence of IL-17RA there is a disruption in the balance of inflammatory mediators leading to aggravated inflammation. This further substantiates that IL-17RA signaling is required for dampening the inflammatory response during HNI-induced OM.

### IL-1 Contributes to Neutrophil Accumulation in the Absence of IL-17RA

**Figure 6:**
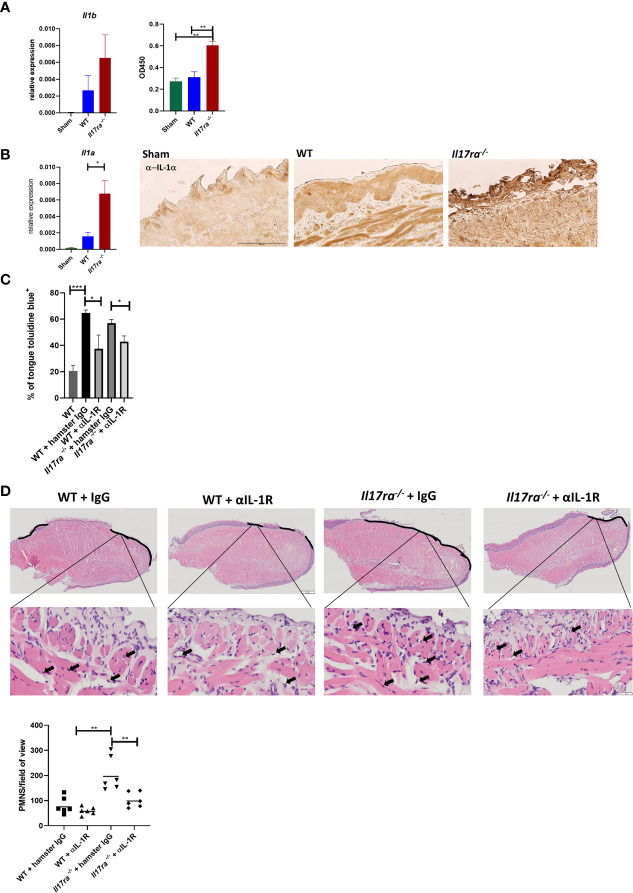
Neutralization of IL-1R alleviates damage in the absence of IL-17RA. **(A)** Gene expression and circulating levels of IL-1β both analyzed by ANOVA with Tukey’s. For gene expression data pooled from at least three experiments. Protein expression represents one experiment. **(B)** Gene expression and IHC of IL-1α pooled from at least three independent experiments. Scale bar, 100 μM. Analyzed by ANOVA with Tukey’s. **(C)** Mice were treated i.p. with αIL-1R at 300 µg on days 0, 2, 4, 6, and 8 (n = at least four). Control animals received appropriate isotype controls (**Supplementary Table 2**). Data represents one experiment. Tongues were harvested and damage quantified by toluidine blue+ staining on D11. Quantification of damage analyzed by ANOVA with Tukey’s *post hoc*. **(D)** Staining by hematoxylin and eosin of sectioned tongues and quantification of neutrophils on day 11 following HNI in the ulcer boundary area. Images captured at 100 μM and zoomed in at 50 μM. Data analyzed by ANOVA with Tukey’s *post hoc*. Data shown as means ± SEM, (*P < 0.05, **P < 0.01, ***P < 0.001).

Since neutrophils were still recruited into the OM lesion even in the absence of IL-17RA, we next determined what inflammatory mediators were responsible for the enhanced neutrophil response in the *Il17ra^−/−^* mice. In WT mice both *Il1a* and *Il1b* are induced post-HNI (**Supplementray Figure 2C**) In the *Il17ra^−/−^* mice there were elevated transcript and protein levels of both IL-1α and IL-1β on Day 11 (**Figures 6A, B**). Since both IL-1α and IL-1β are implicated in the pathology of OM, and IL-1α at least partially regulates neutrophils in other oral diseases (33, 51–53), we asked if blocking IL-1R in WT and *Il17ra^−/−^* mice would account for the increased damage when IL-17RA is lacking. Following α-IL-1R Ab administration, OM damage was decreased in WT (~1.8-fold reduction) and *Il17ra^−/−^* mice (~1.3-fold reduction) compared to mice administered hamster IgG isotype control Abs (**Figure 6C**). Of note, the mice that received isotype control Abs had 3-fold higher damage compared to radiated mice that did not receive control Abs. This may be due to species specificity, antibody concentration or the dosing schedule since mice that received mouse IgG1 control Ab at a lower dose and less frequently did not present with increased tongue damage (**Figure 2C** and **Supplementary Table 2**). Even though mice treated with hamster IgG control antibody showed increased damage in the oral mucosa after radiation it was not related to elevated neutrophil numbers within the area of ulceration (**Figure 6D**). Yet, in irradiated *Il17ra^−/−^* mice the lessening of overt damage when IL-1R was blocked correlated with a decrease in neutrophils in the areas of ulceration (**Figure 6D**). This substantiates that when IL-17RA signaling is missing uncontrolled production of IL-1 is at least partially responsible for the increased damage and enhanced neutrophil response, and that α-IL-1R is a viable treatment option to explore in OM.

### Neutrophil Blockade Accounts for the Excess Damage in the Absence of IL-17RA

**Figure 7:**
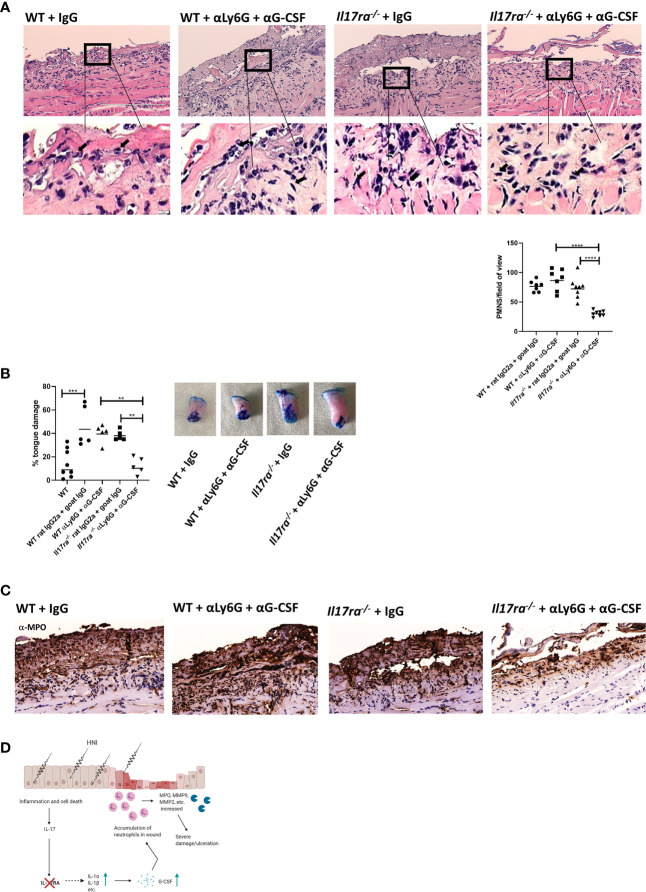
Neutralization of neutrophils reduces damage in the absence of IL-17RA. Mice were treated i.p. with αLy6G at 150 µg and αG-CSF at 10 µg (n = 5/group) on day 7 following radiation. On days 8–10 mice were treated with 10 µg of αG-CSF. Control animals received appropriate isotype controls (**Supplementary Table 2**). Data is representative of two independent experiments. **(A)** Staining by hematoxylin and eosin of sectioned tongues and quantification of neutrophils on day 11 following HNI. Images captured at 200 and 10 μM. Data analyzed by ANOVA with Tukey’s *post hoc*. **(B)** Tongues were harvested and damage quantified by toluidine blue+ staining on D11. Data analyzed by ANOVA with Tukey’s. **(C)** Representative MPO-staining of ulcer boundary counterstained with hematoxylin. **(D)** Proposed mechanism of IL-17RA-mediated protection and healing of OM. Radiation induces inflammation and apoptosis, increasing IL-23 expression. IL-23, in turn activates IL-17RA-mediated inflammation. In the absence of IL-17RA, IL-23, along with IL-1a synergizes to induce excess neutrophil chemokines like G-CSF resulting in accumulation of neutrophils in the damaged area. Increased neutrophils release proteases that further damage the wound and contribute to delayed wound healing. Image created using BioRender with permission. Data shown as means ± SEM, (**P < 0.01, ***P < 0.001, ****P < 0.0001).

To further establish the pathogenic consequence of an elevated neutrophil response in the absence of IL-17RA we used a combination α-Ly6G and α-G-CSF or rat IgG2a and goat IgG isotype controls in WT and *Il17ra^−/−^* mice (33). As in the IL-1R blockade study (**Figure 6C**), rat IgG2a and goat IgG administration resulted in increased damage compared to irradiated untreated mice, and again this higher damage was not related to an increased neutrophil influx (**Figure 7B**). Yet, α-Ly6G/G-CSF treatment resulted in the depletion of the excess neutrophils in *Il17ra^−/−^* mice (**Figure 7B**). This was associated with a reduction in tongue damage of around 2.5-fold (**Figure 7A**) and lower MPO activity (**Figure 7C**). The OM damage in WT mice was unaffected though following neutrophil depletion compared to mice that received isotype control Abs using this particular method and dosing schedule. Collectively these data suggest that IL-17RA is critical for modulating the neutrophil response during HNI, and in the absence of IL-17RA, IL-1-mediated neutrophil accumulation exacerbates inflammation and is detrimental to healing (**Figure 7D**).

## Discussion

While robust models exist to study damage in the skin, highly tractable systems to study injury of the oral mucosa have been lacking. Infections that breech the mucosal barrier in the oral cavity require healing and restoration of the epithelium, but the injury is not as easily followed to the healing phase (54). To understand the immune components involved in repair processes in the oral mucosa, a model with a strong readout of damage (i.e. severe ulceration) that heals with predictable kinetics is required. We used a single dose of radiation that allowed for the development of OM that could be assessed visually and histologically, but also allowed procedural ease when using a clinical linear accelerator.

In general the pathogenesis of OM is similar between mice and humans (8). In patients, OM lesions can be detected on most keratinized tissues in the mouth that are exposed to radiation, including the tongue, buccal mucosa and the soft palate. Interestingly, overt damage was localized to the distal portion of the tongue in all mice (**Supplementary Figure 1B**). Also, IL-17RA-related gene targets were more strongly activated where the lesions predominated in WT tongue, and we postulate this is the region of the tissue that requires IL-17 to heal properly (**Supplementary Figure 1B**). The location of damaged tissue in the mouse could be an artifact of the way the mice are arranged in the apparatus of the linear accelerator or how the head and neck region is being exposed to radiation. In addition, the architecture of the mucosal surfaces in the oral cavity do vary between mice and humans, evidenced by distinct keratinization and cellular distribution patterns, which may explain the distribution of lesions in mice (55). We also have to consider the distribution of the cytokine receptors throughout the supra- and sub-basal epithelial layers of the oral mucosa. While IL-17RA is expressed ubiquitously, it is the expression of IL-17RA in the supra-basal epithelia that is required for protection from OPC (56), whereas mice deficient in IL-17RA specifically in the superficial epithelia (*Il17RA* ^fl/fl^-K13^Cre^) are as susceptible to OPC as IL-17RA knockout mice. Therefore, follow-up studies using tissue-specific knockout mice are justified and will start to dissect these relationships.

IL-17 is highly expressed in ulcerated areas in a WT mouse and the overall effect of IL-17RA-signaling is beneficial during OM since *Il17ra^−/−^* mice showed considerably more damage. This is not the role attributed to the cytokine in other forms of recurring oral ulcers associated with leukocyte adhesion deficiency type 1 (LAD1), where both IL-23 and IL-17 are pathogenic (57). Now we establish that IL-17RA is necessary post-irradiation for maintaining the epithelial barrier through modulation of pro-apoptotic factors, while promoting effector molecules involved in cell survival and proliferation. This is similar to how IL-17RA helps maintain barrier integrity in the gastrointestinal tract (23). Furthermore, a striking phenotype of the *Il17ra^−/−^* mice was an unexpected increase in neutrophils found at the site of ulceration compared to WT mice 11 days post-irradiation. Normally IL-17 promotes an influx of neutrophils that is either protective or pathogenic depending on disease context through induction of neutrophil chemokines CXCL1,2 and 5 and growth factors such as G-CSF (58). In this way, mice deficient in IL-17 signaling components (*Il17a^−/−^*, *Il17ra^−/−^*, *Act1^−/−^* etc.) are particularly sensitive to infections in which prevention is dependent on the action of neutrophils, such as oral candidiasis (59). Inversely, these same knockout mice are at least partially protected from autoimmune disease through a dampened neutrophil response (60). Yet, during HNI-induced OM, expression of neutrophil chemokines was not diminished in *Il17ra^−/−^* mice. In fact, *Csf3*, which encodes for G-CSF, was significantly enhanced in the absence of IL-17RA. Concomitantly, IL-1α and IL-1β were overproduced in *Il17ra^−/−^* mice compared to WT. The dysregulated IL-1 levels in the absence of IL-17RA appear partially responsible for the elevated PMNs since blocking of IL-1R led to a reduction, but not a complete loss, of neutrophilic cells in the lesion and lessening of OM-mediated damage. The interplay between IL-1 and IL-17 in OM does align with the role of these cytokines in other oral diseases including OPC, during which IL-1α in part controls the neutrophil response (33).

Prolonged exposure to neutrophils can lead to enhanced tissue damage. This is because neutrophils secrete a repertoire of proteases and enzymes such as MMPs, elastase, Cathepsin G, and MPO that damage tissue (61, 62). MPO is abundantly expressed in neutrophil granules and is associated with severe OM lesions (45, 63) and delayed wound healing (64). Neutrophils also produce various MMPs, such as MMP-9, which was detected at elevated levels in *Il17ra^−/−^* mice, and is associated with tissue destruction during periodontal disease and OM (65, 66). While the action of MMPs triggered by DNA damage associated with ionizing radiation contributes to the ulceration of the oral mucosae, MMPs are also required to breakdown the ECM for healing. Therefore, regulation of this system is critical (67, 68). The pathogenic nature of excess PMNs in *Il17ra^−/−^* mice was substantiated by the efficacy of α-Ly6G/-G-CSF treatment that greatly reduced neutrophils in the lesion and led to a reduction in damage. Although, this therapy did not result in a reduction of PMNs or damage in WT mice. We speculate this could be due to the treatment schedule used, and modifications in dosing may result in the blocking of neutrophils in WT mice and therapeutic efficacy. Indeed, antibody-based depletion of neutrophils can be complicated (69). Another possibility is since expression of *Csf3* was markedly increased in *Il17ra^−/−^* mice, targeting G-CSF was particularly successful in these mice. Future studies to determine the potential of neutrophil blocking treatment in IL-17RA sufficient mice are warranted.

It is also important to consider that although oral ulcers in WT and *Il17ra^−/−^* mice started to heal at the same time, peak damage was more severe in mice deficient in IL-17RA (**Supplementary Figure 1** and data not shown). This has important implications for patients that develop OM, since often it is the severity of disease that dictates termination or alteration of cancer therapies (2). It is critical to consider how the function of IL-17 in OM could also complicate targeting the Th17/IL-17 pathway to treat the associated malignancies. Because of the tumorigenic properties of IL-17 in the head and neck, it follows that the blockade of these cytokines in combination with other therapeutics might be efficacious in treating malignancies (70). However, our findings would indicate that anti-IL-17 therapy exacerbates OM symptoms, complicating the therapeutic benefit of this strategy to fight head-neck cancers.

Our finding that treatment with α-IL-17A antibody rendered mice as susceptible to OM as *Il17ra^−/−^* mice, may have implications for individuals receiving therapies that block Th17/IL-17 related immune components. Indeed, a rare case of lichenoid mucositis has been reported with secukinumab (α-IL-17A monoclonal antibody) use (71). As the administration of anti-IL-17-related treatments increase, our study indicates that these patients should be monitored for development of mucositis. Susceptibility to severe OM is partly dependent on patient factors such as smoking and overall oral health at the start of treatment, while the genetic predispositions related to the incidence of OM are less understood. Genome-wide association studies have identified genomic loci pathways related to RT-induced OM, yet no associations with the Th17 cells or IL-17 have been identified (72). Further genomic studies considering other forms of cancer therapies may elucidate a role for IL-17 and other inflammatory pathways in OM and lead to better personalized treatment regiments (73).

In conclusion, we provide evidence that IL-17RA is important for protection during HNI-induced OM (**Figure 7C**). We propose that IL-17RA is critical for preventing excessive inflammation during the ulceration phase of OM. In the absence of IL-17RA other cytokines and chemokines that are in abundance amplify immune cell migration into the oral mucosae creating an environment of excessive inflammation and damage in the mucosal layer. Further, without IL-17RA a reduced proliferative capacity of the epithelium, along with increased apoptosis, leads to a breakdown in the maintenance of the mucosal layer. IL-17RA is necessary to prevent severe damage and promote healing after ionizing radiation, and this important contribution has implications for cancer therapies related to the Th17 pathway.

## Data Availability Statement

The datasets presented in this study can be found in online repositories. The names of the repository/repositories and accession number(s) can be found below: BioProject PRJNA720631, https://www.ncbi.nlm.nih.gov/bioproject/PRJNA720631.

## Ethics Statement

The animal study was reviewed and approved by the University of Toledo IACUC.

## Author Contributions

Conceptualization, Funding Acquisition and Projec Administration, HC. RNA-Seq computational analysis, VB, ACS, CM. FACS analysis, DL. Head-neck irradiation, EP and AL. Histology, AS, JS-M and JD. Experimental Support, JS-M, JD, JK, DL, EA, AW, GM, PB, and SC. Experimental Design, HC and JS-M. Paper writing, JS-M, EP and HC. All authors contributed to the article and approved the submitted version.

## Funding

HC is supported for this work by a seed grant from Ohio Cancer Research and VB is supported by U19AI110820.

## Conflict of Interest

The authors declare that the research was conducted in the absence of any commercial or financial relationships that could be construed as a potential conflict of interest.

## Supplementary Material

Supplementary Figure 1 WT mice exposed to radiation develop predictable OM stages **(A)**. WT mice exposed to 22.5 Gy develop OM damage that peaks on Day 11 and is cleared by day 15. Data analyzed by ANOVA with Tukey’s and represents at least 3 experiments **(B)**. Solid lines depict the boundaries for considering what portion of the dorsal (top) tongue a lesion was found: proximal (near the excision-site), middle, or distal (tip of the tongue). Dashed line indicates where the tissue was portioned for RNA-seq analysis. Lesion location distribution in **(C)** WT mice (n=51) and **(D)** *Il17ra* -/- mice (n=20). ​ Data shown as means +/- SEM, (*P<0.05, **P<0.01,****P<0.0001).

Supplementary Figure 2 Changes in expression of IL-24 after exposure to HNI. Expression of *Il24* on D11 following radiation. Data pooled from at least 3 experiments and analyzed by ANOVA with Tukey’s. Representative IHC images of IL-24 protein expression. Unfilled arrows represent negative staining while filled arrows represent positive staining. **(C)** Expression kinetics of *Il1a *and *Il11b *following HNI assessed by qPCR. Results pooled from at least 3 experiments. Data shown as means +/- SEM, (*P<0.05, **P<0.01,****P<0.0001).

Supplementary Figure 3 Gating strategy. **(A)** Gating strategy for neutrophils. **(B)** Gating strategy for necrosis, early apoptosis, and late apoptosis.
