# Supplementary material for: Tissue Damage in Radiation-Induced Oral Mucositis Is Mitigated by IL-17 Receptor Signaling
Source: Front Immunol. 2021 Jun 17;12:687627. doi: 10.3389/fimmu.2021.687627 (PMC8248500; doi:10.3389/fimmu.2021.687627)
Supplement: Supplementary Figure 1 — WT mice exposed to radiation develop predictable OM stages (A). WT mice exposed to 22.5 Gy develop OM damage that peaks on Day 11 and is cleared by day 15. Data analyzed by ANOVA with Tukey’s and represents at least 3 experiments (B). Solid lines depict the boundaries for considering what portion of the dorsal (top) tongue a lesion was found: proximal (near the excision-site), middle, or distal (tip of the tongue). Dashed line indicates where the tissue was portioned for RNA-seq analysis. Lesion location distribution in (C) WT mice (n=51) and (D) Il17ra -/- mice (n=20). ​ Data shown as means +/- SEM, (*P<0.05, **P<0.01,****P<0.0001). [file Presentation_1.pptx]

## Slide 1
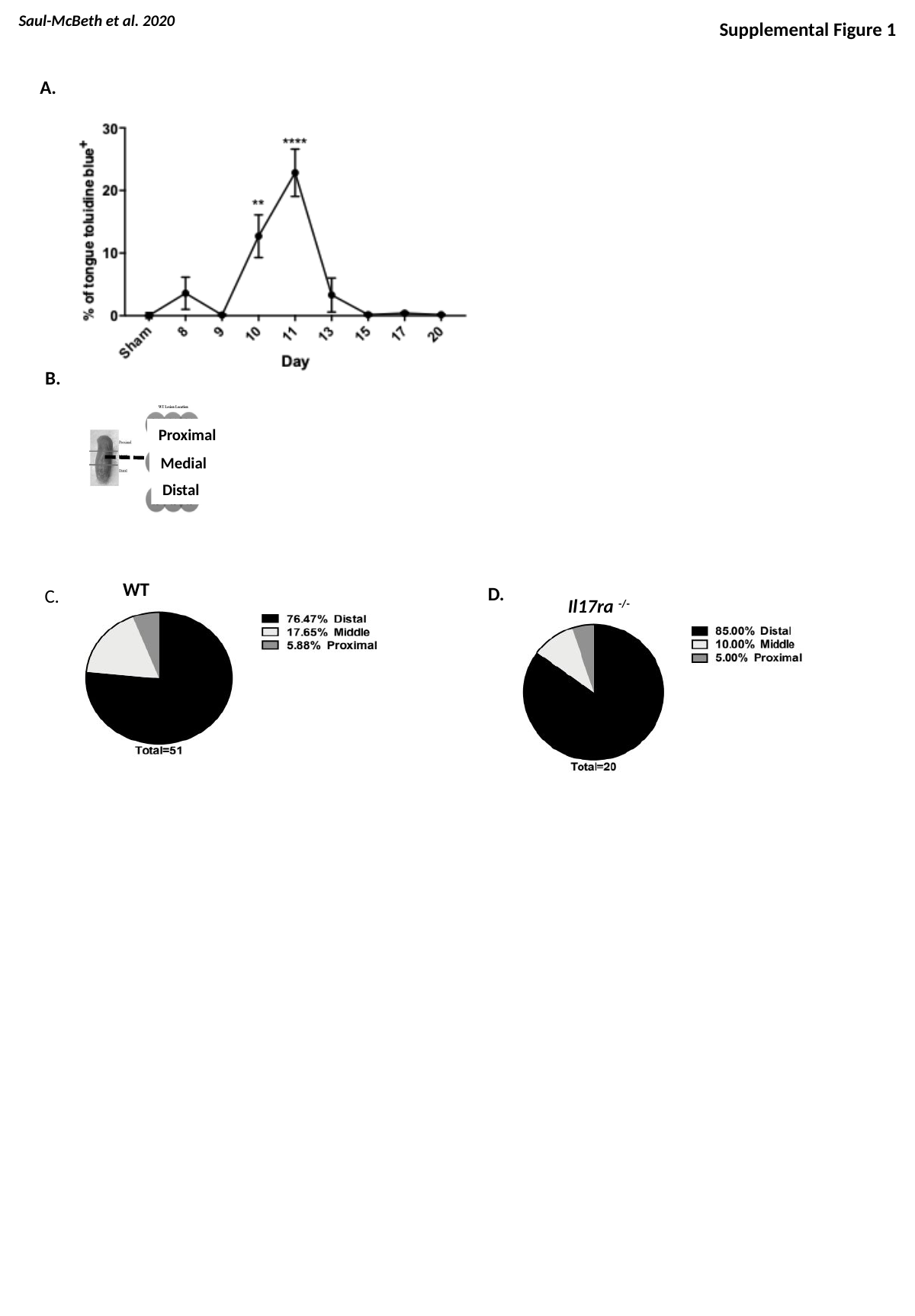

Saul-McBeth et al. 2020
Supplemental Figure 1
A.
B.
Proximal
Medial
Distal
WT
D.
C.
Il17ra -/-

## Slide 2
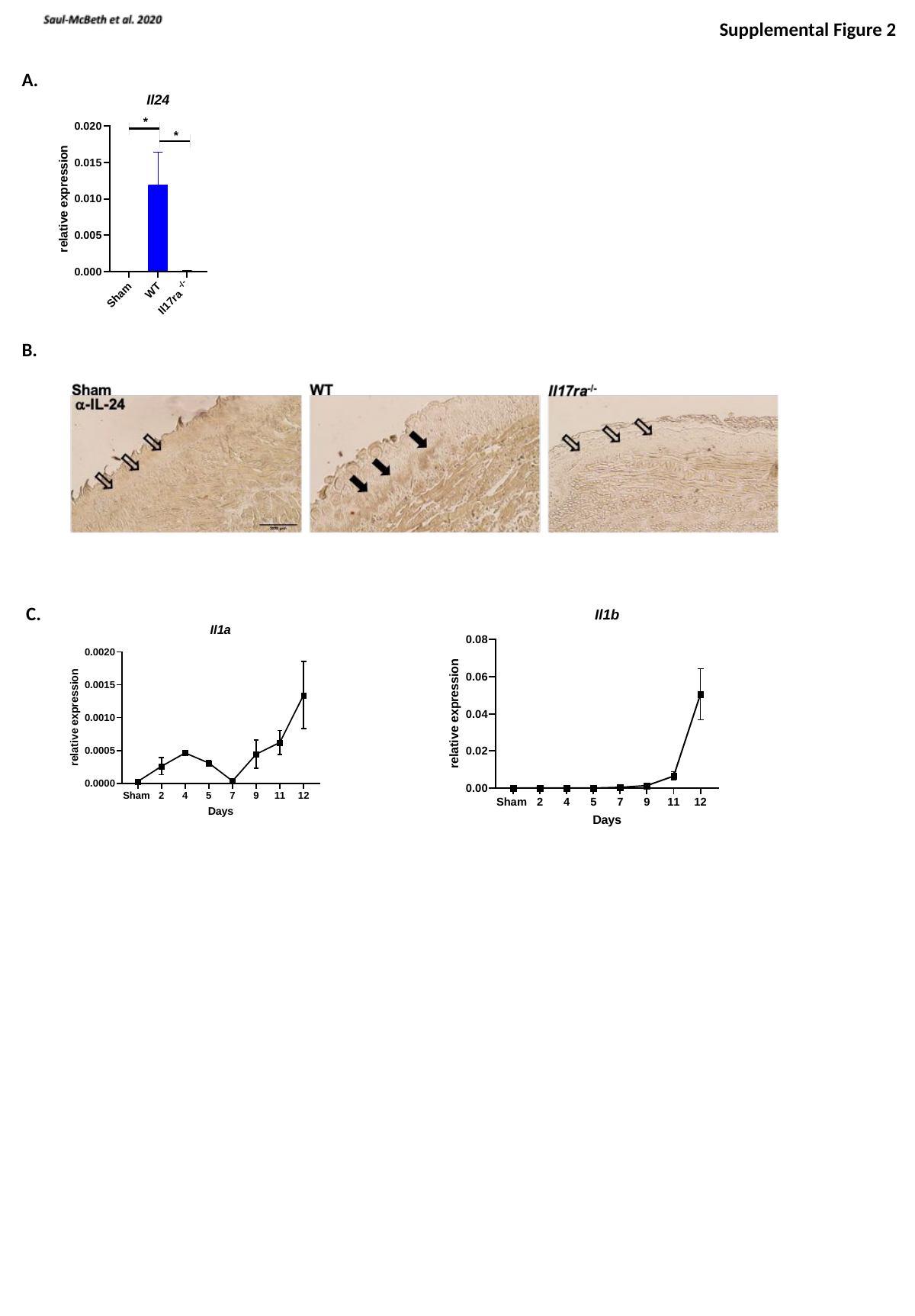

Supplemental Figure 2
A.
B.
C.

## Slide 3
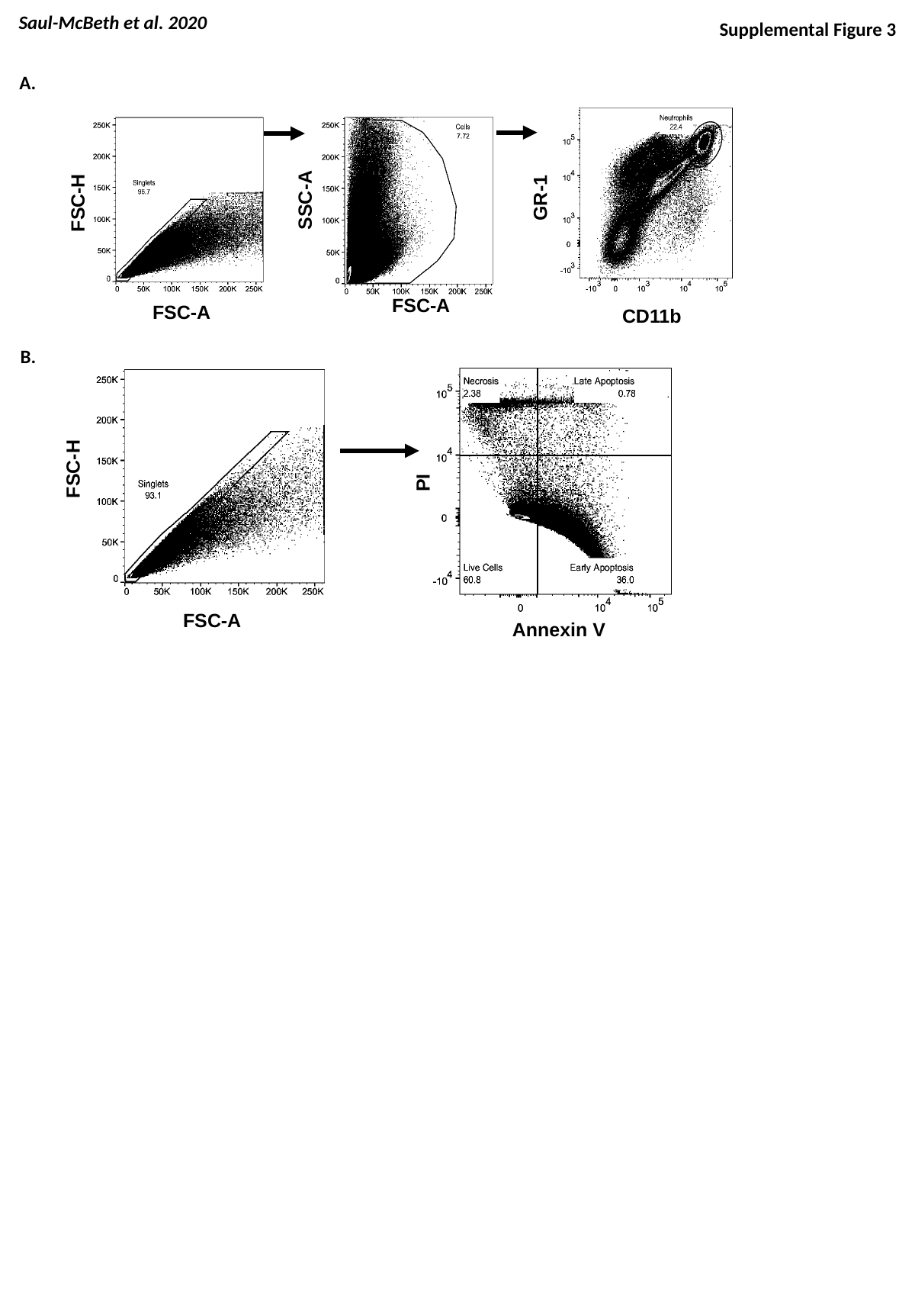

Saul-McBeth et al. 2020
Supplemental Figure 3
A.
FSC-H
FSC-A
SSC-A
FSC-A
GR-1
CD11b
SSC-A
CD11b
B.
FSC-H
PI
FSC-A
Annexin V

## Slide 4
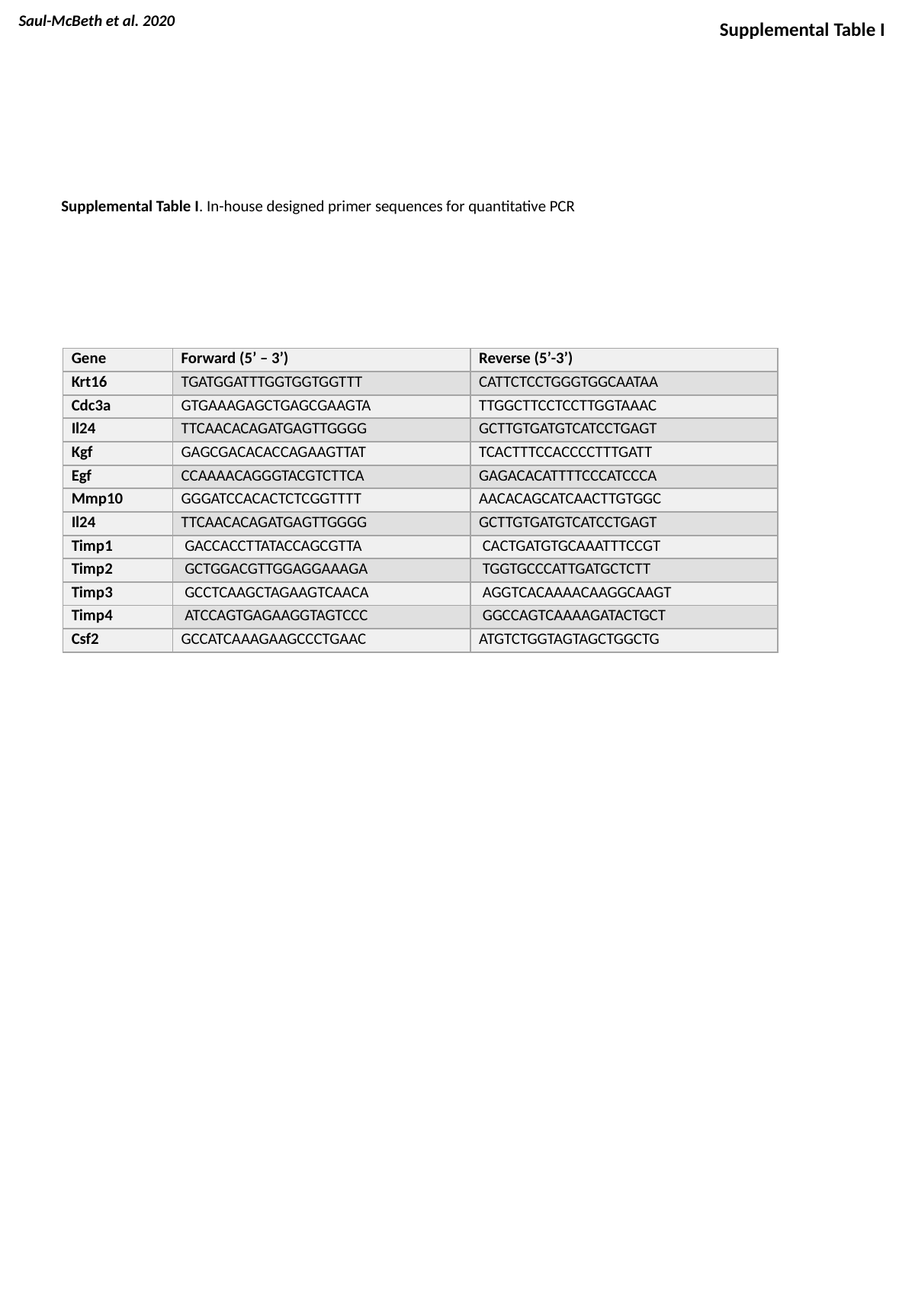

Saul-McBeth et al. 2020
Supplemental Table I
Supplemental Table I. In-house designed primer sequences for quantitative PCR  ​
| Gene | Forward (5’ – 3’) | Reverse (5’-3’) |
| --- | --- | --- |
| Krt16 | TGATGGATTTGGTGGTGGTTT | CATTCTCCTGGGTGGCAATAA |
| Cdc3a | GTGAAAGAGCTGAGCGAAGTA | TTGGCTTCCTCCTTGGTAAAC |
| Il24 | TTCAACACAGATGAGTTGGGG | GCTTGTGATGTCATCCTGAGT |
| Kgf | GAGCGACACACCAGAAGTTAT | TCACTTTCCACCCCTTTGATT |
| Egf | CCAAAACAGGGTACGTCTTCA | GAGACACATTTTCCCATCCCA |
| Mmp10 | GGGATCCACACTCTCGGTTTT | AACACAGCATCAACTTGTGGC |
| Il24 | TTCAACACAGATGAGTTGGGG | GCTTGTGATGTCATCCTGAGT |
| Timp1 | GACCACCTTATACCAGCGTTA | CACTGATGTGCAAATTTCCGT |
| Timp2 | GCTGGACGTTGGAGGAAAGA | TGGTGCCCATTGATGCTCTT |
| Timp3 | GCCTCAAGCTAGAAGTCAACA | AGGTCACAAAACAAGGCAAGT |
| Timp4 | ATCCAGTGAGAAGGTAGTCCC | GGCCAGTCAAAAGATACTGCT |
| Csf2 | GCCATCAAAGAAGCCCTGAAC | ATGTCTGGTAGTAGCTGGCTG |

## Slide 5
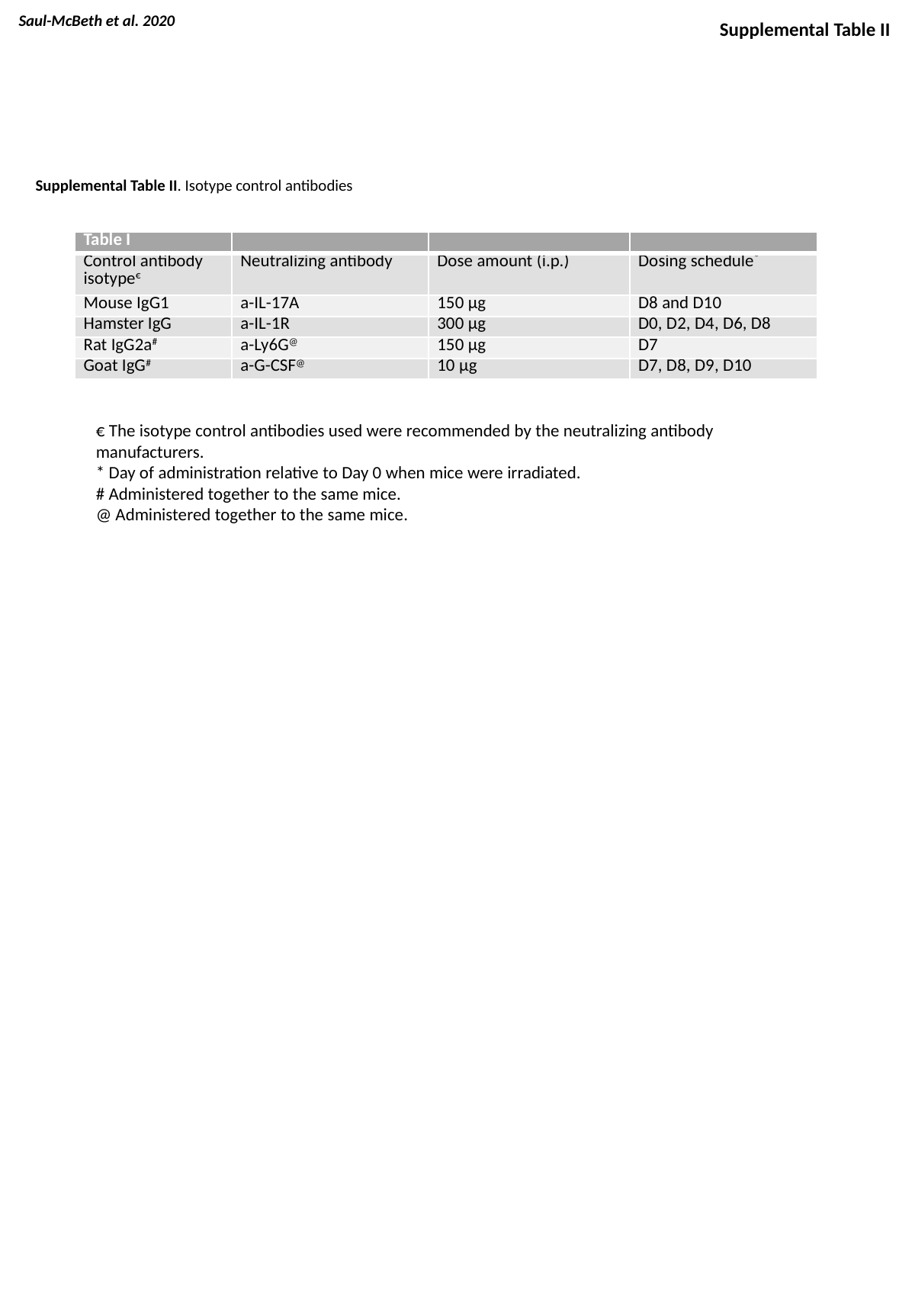

Saul-McBeth et al. 2020
Supplemental Table II
Supplemental Table II. Isotype control antibodies ​
| Table I | | | |
| --- | --- | --- | --- |
| Control antibody isotype€ | Neutralizing antibody | Dose amount (i.p.) | Dosing schedule\* |
| Mouse IgG1 | a-IL-17A | 150 μg | D8 and D10 |
| Hamster IgG | a-IL-1R | 300 μg | D0, D2, D4, D6, D8 |
| Rat IgG2a# | a-Ly6G@ | 150 μg | D7 |
| Goat IgG# | a-G-CSF@ | 10 μg | D7, D8, D9, D10 |
€ The isotype control antibodies used were recommended by the neutralizing antibody manufacturers.
* Day of administration relative to Day 0 when mice were irradiated.
# Administered together to the same mice.
@ Administered together to the same mice.
